# How threats inform conservation planning—A systematic review protocol

**DOI:** 10.1371/journal.pone.0269107

**Published:** 2022-05-31

**Authors:** Elina A. Virtanen, Maria Söderholm, Atte Moilanen

**Affiliations:** 1 Finnish Natural History Museum, University of Helsinki, Helsinki, Finland; 2 Marine Research Centre, Finnish Environment Institute, Helsinki, Finland; 3 Information Services, Finnish Environment Institute, Helsinki, Finland; Institute of Geographic Sciences and Natural Resources Research Chinese Academy of Sciences, CHINA

## Abstract

Conservation planning addresses the development and expansion of protected areas and requires data on for instance species, habitats, and biodiversity. Data on threats is often minimal, although necessary in conservation planning. In principle, threats should guide which conservation actions to take and where, and how to allocate resources. The lack of threat information may also limit the validity of areas to be conserved, if the condition of areas is degraded by threats unknown. The protocol described here outlines the methodology for a systematic review to explore how threats are theoretically and methodologically understood and used in conservation plans across freshwater, marine and terrestrial environments. Our primary research question is: how have threats informed conservation planning? Studies will be categorized according to the types of threats and conservation features used, theoretical and methodological approaches applied, geographical context, and biome. The results are expected to increase our understanding about how threats can and should be addressed in conservation planning.

## Introduction

Protected areas are the backbone of biodiversity conservation. Recent biodiversity targets, set out for instance by the EU Biodiversity Strategy, aim to expand conservation area coverage to 30% by the end of 2030 to halt biodiversity loss [1]. The effectiveness of such targets depends on whether we are protecting the “right” places, such as areas with high biodiversity or ecological importance [2–5]. The subfield of conservation biology, systematic conservation planning (SCP), aims to bring most important areas under protection and to guide restoration and management efforts to locations where benefits for biodiversity are obtained in a cost-effective manner [6, 7]. SCP usually addresses the simultaneous protection of hundreds or thousands of conservation features, such as species, habitat types, ecological communities, ecosystems, ecosystem services or even ecological functions [8, 9].

Achieving conservation targets requires also knowledge about the threats (pressures, stressors, drivers) facing biodiversity that may undermine conservation action [10–13]. The intensity, frequency, and severity of human-induced threats to biodiversity vary through space and time [14], and the resulting impacts depend on the vulnerability and recovery potential of species [15, 16]. The utility of conservation plans depends on the quantity and quality of data included, which may be rich in biodiversity features but poor in threat data [8]. Threats are in conservation planning known less well and used less frequently, even when they are the underlying reason why biodiversity conservation is needed in the first place.

In SCP, threats typically guide conservation away from areas where significant threats are present. Such areas may be further degraded making them undesirable for the development of protected areas. Conservation may also be easier and cheaper in environments not occupied by people and thus under lower pressures [17], although conservation may be more successful by engaging local communities [18]. Threats may also attract conservation interest if they are stoppable (abatable): for example, overfishing can be alleviated by regulation. Stoppable threats are (almost) always local, making it difficult to map them over broad geographical areas through time [10]. This hinders our capacity to develop effective conservation measures. Proxies for threats are often used instead, including such as land-use changes over time [19] or the spatial footprint of human activity [20].

Determining the impacts of threats on species or ecosystems is a separate process in conservation assessments. Understanding the spatial and temporal patterns of threats provides a basis for mitigating environmental damage in sensitive areas [12]. Maps of the negative impacts of human activities have been developed for freshwater, marine and terrestrial realms [21–23]. While broadly useful for separating areas based on the levels of anthropogenic presence, the fundamental drivers of the impacts may remain unknown. Also, the ecological responses to conservation actions taken may be partially unknown. Consequently, it is not trivial to identify actions that are cost-effective in the combatting of threats.

Here, we aim to understand how threats have been used in conservation planning research. More specifically, we aim to synthetize how threats have been defined, how they have been used, and how they have informed conservation planning. We define threats following Salafsky et al. 2008 [24] as: “The proximate human activities or processes that have caused, are causing, or may cause the destruction, degradation, and/or impairment of biodiversity targets”. We will follow the threat classification scheme of the IUCN Red List (version 3.2). This work will build on previous studies that have mapped threats to species [25] and human pressures on biodiversity [26], assessed threats in terrestrial conservation areas [27], evaluated the effectiveness of protected areas at resisting anthropogenic threats [28] or reviewed threats to biodiversity in the future [29]. A large number of studies mention the importance of combating threats for conservation. A much smaller number of studies either map or model threats to individual species or habitats.

This protocol describes the methodology that a systematic review will follow. The proposed work will provide an overview of literature describing threats in the context of conservation planning. The methodological and theoretical extent of existing studies will be evaluated. To our knowledge, no systematic review of threats in conservation planning has been accomplished. Hence, there is potential for generating new theoretical and methodological insights, as well as guidelines from studies that have previously been disconnected in the absence of a unified conceptual framework. Our systematic review will organize existing information about the use of threats in conservation plans, and potentially will develop new principles for the use of threat information in conservation planning. The results will also improve our understanding of how threats could be reported systematically in conservation planning. Such methodological findings would advance the field of systematic conservation planning and benefit the conservation planning community at large.

The primary research question for the review is: How have threats informed conservation planning? Secondary research questions are shown in Table 1. This study will include conservation plans that have used threats at any spatial scope (local, national, continental, global) or in any realm–terrestrial, marine, or freshwater (or combination). Conservation plans can target individual species, habitats, biotopes, ecosystems, or important areas that need protection, such as high-biodiversity, key ecological or essential biodiversity areas. Reviewed literature may cover the past, present or future. The topic may be design of protected areas under changing environmental conditions or conservation effort under alternative management scenarios. Studies may also include methodological and theoretical approaches if relevance for conservation planning is described.

**Table 1:** Secondary research questions for the systematic review.

1. What is the geographic distribution, spatial resolution, and scale of studies?
2. Which biodiversity features (species, habitats, ecosystems, etc.) the studies represent?
3. Has the sensitivity of biodiversity features to threats been established, and if yes, how?
4. Which threats and how many of them have been included?
5. How has the threat data been generated?
6. What are the methodological approaches for including threats in conservation planning?
7. Are threats connected individually to biodiversity features or are threats used in an aggregate manner?
8. How have temporal aspects of threats been addressed in the studies? Does the study concern the past, present or future?
9. Do the studies report conservation actions to counteract threats?
10. Do the studies also consider the costs of threat abatement?

## Materials and methods

This review protocol follows the guidelines set out by the PRISMA-P (Preferred reporting items for systematic review and meta-analysis protocols) [30] (S1 File) and is amended with the reporting standards of ROSES (Reporting standards for Systematic Evidence Syntheses) [31].

### Search plan

The aim is to compile all relevant scientific literature by using multidisciplinary databases. Development of the search plan included a scoping phase to gain comprehensive understanding of relevant keywords, to perform keyword analysis and selection, as well as define the search plan. The search syntaxes and the results of the scoping phase searches are summarized in S2 File. The scoping phase searches were conducted as a topic search in the Web of Science (WoS) Core Collection. The searches were based on keywords and search phrases initially defined by the research team.

Several test searches were run by combining terms with Boolean operators AND/OR and proximity operator NEAR with varying proximity of keyword combinations to define the keywords, best keyword combinations and to optimize search phrases and strategy. No limitations were placed on the date, language, or type of publication. The first 500 studies of the 1^st^ scoping phase search were evaluated based on eligibility criteria, and 176 were determined to be relevant. The results of the scoping study were compared with the study objectives. Based on the analysis, missing terms were added, and the search strategy was amended with relevant synonyms and additional terms.

Table 2 presents the keywords for the search and the final search strategy will consist of the three-step approach outlined in Table 3. The conservation planning terms used cover general and more specific core terms related to the conservation planning research. However, conservation planning terms are not always used in literature.

**Table 2:** The keywords for literature searches.

Conservation planning terms	conservation plan, conservation planning, conservation prioritization, reserve selection, site selection, spatial prioritization, systematic conservation
Conservation area terms	conservation area, protected area
Threat and related terms	threat, pressure, stressor, anthropogenic, risk, impact
Planning terms	planning, plan, design, designing
Conservation terms	conservation, conserving, protection, protecting, preservation

**Table 3:** Search steps and phrases based on keywords.

Step 1	Conservation planning terms AND threat and related terms
Topic: Conservation planning	
Step 2	Conservation area terms AND threat and related terms AND planning terms
Topic: Conservation area	
Step 3	Site selection AND threat and related terms AND planning OR conservation terms
Topic: Site selection	

Both threat-related and planning-related terms are essential to narrow search results down to relevant conservation literature. Threat terms were used in basically all searches and combined with other search terms using Boolean operators. Planning-related terms, on the other hand, are used to retrieve the most accurate results possible in searches where the core keywords do not unambiguously identify the study within the conservation planning literature. The terms "conservation area", "protected area" and "site selection" were identified as such terms in the scoping stage test searches. In addition, the test searches with the term “site selection” showed that a search with planning terms alone is not sufficient. Thus, the scope of that search was broadened by adding the conservation terms to the search, ensuring that we do not miss publications that may meet the eligibility criteria set for publications. The S3 File sums up the keywords and search steps as well as the plan for search protocol and the final query strings of the study.

The aim of the first search step with the most typical keywords for conservation planning and narrower concepts (e.g., conservation prioritization), is to broadly cover conservation planning literature that deals with threats. This step forms the core of the search strategy and is expected to capture the main body of potentially relevant research literature. The second step “Conservation area” will find studies that concern both threats and planning. This is achieved by utilizing Boolean syntax to combine both "threat" and "planning" terminology in the query string. The third search step is based on term “site selection” that is not fully specific to conservation research but can also be used in other fields, including selection of industrial sites. The search steps identify literature that, according to the scoping phase results, is broadly relevant for the present work. The aim of the third step is to return literature limited to threats in the context of conservation. The final collection of studies for screening and extraction will consist of search results combined with duplicates removed.

The search strategy will be implemented as uniformly as possible in all databases. However, there are some differences in the technical options available at different services, so the execution of the search strategy must be fitted accordingly. Topic search is utilized in reference databases to focus the search on the title, abstract, and keyword terms of the publication. When topic search is not available, we will do our best to target the search to all publication-related information as available on the service. For example, the analysis of the scoping phase results changed the search performance in the WoS database. In the WoS database the topic search will be replaced with a combination of title, abstract, and author keyword search to avoid the KeyWords Plus index terms that are automatically generated from the titles of cited articles and lead to non-eligible results.

### Bibliographic databases and data management

A structured search in English will be conducted in the Scopus and the Web of Science (Core Collection), targeting academic literature including peer-reviewed research articles and conference proceedings. Because the review topic is quite broad, additional sources of information, such as organizational websites, will not be considered. Grey literature will be excluded from the search, to preserve the global comparability between the studies. We acknowledge that national conservation plans are most likely published as grey literature in national languages, but such studies are out of scope for this review.

The database searches in the Web of Science and Scopus to retrieve and analyse the publication information will be done under the existing subscriptions of the organizations of the authors. Most full-text publications will be immediately available by the organizational subscriptions, and the rest will be obtained through library services. Search results will be downloaded and catalogued in the EndNote X9 version. Article importing and data manipulation will be done with the support of the R package revtools [32].

### Estimating the comprehensiveness of the search

We defined a list of 12 publications, based on the results of the scoping phase. These publications were used as a benchmark to assess the comprehensiveness of our search phrases and to develop our search procedure as necessary (S4 File). The selection of articles was based on the study eligibility criteria. The search strategy aims to bring all relevant studies, using the two-stage approach, where the main collection will be compiled using broader search terms and complementing it with narrower terms. Searches conducted according to the search plan of S3 File showed that all articles in the benchmark list (S4 File) would have been captured by almost all search steps. Thus, relevant literature is easily included in the final literature database for screening and extraction.

### Article screening and study inclusion criteria

#### Screening strategy

Titles and abstracts will be screened with the support of R package revtools [32] according to the predefined inclusion criteria (see below). The title and abstract must clearly state that the study concerns conservation planning and claims to have included threats in the planning process. It is expected that threat information is not necessarily mentioned in the abstract, as most studies concentrate on reporting the findings of conservation plans. If there is insufficient information for a study to be conclusively excluded based on the predefined eligibility criteria, it will be forwarded to the next phase. In the full-text phase, all studies excluded and the reasons for exclusion will be catalogued for transparency.

#### Inclusion criteria

Eligible studies must deal with conservation planning and threats. Studies may encompass any biome (terrestrial, freshwater, marine, or combination). They may cover any geographical region or temporal period, including historical or future conservation plans. Studies which do not relate to any specific target of biological conservation feature, will be excluded. All methodological approaches to the design of conservation areas will be included, to allow a comprehensive evaluation of how conservation outcomes are informed about threats. Purely methodological papers will be considered if the applicability of the methods is tested and shown. It is expected that resource-limited conservation interventions may not necessarily use quantitative tools, such as decision-support frameworks or GIS assisted planning. Instead, the planning of conservation areas may rely on expert-based information, and such studies will also be included. Detailed inclusion and exclusion criteria are shown in Table 4.

**Table 4:** Inclusion and exclusion criteria for screening.

**Inclusion criteria for title and abstract screening:**
a) The study must be about conservation planning
b) The study must include threats (if impossible to define based on the abstract, studies will be forwarded to full-text screening)
**Inclusion criteria for full-text screening:**
a) Threats are used in conservation planning, and
b) Threats are human-driven or caused by a human-initiated process, and
c) the aim of the study is ecological
**Exclusion criteria for full-text screening:**
a) Threats are not used in the development of the conservation area network.
b) The threat is caused by a natural hazard (e.g. geological events)
c) The study is only a theoretical/methodological work, and does not report the direct applicability of the approach to conservation planning

#### Consistency checking

The article screening will mainly be carried out by one reviewer. At each screening phase, a random set of studies (10%, or a minimum of 50) will be validated by a second reviewer. The studies included will be compared to determine whether the two reviewers are in an agreement with respect to the inclusion criteria. Cohen´s Kappa statistics [33] will be calculated for the (dis)agreement. Should the results differ significantly (<0.6), the inclusion criteria will be further clarified, disagreements discussed, and eligibility criteria reformulated accordingly.

### Data extraction

Extraction of data from full-text screened articles will be completed by filling in a pre-coded data sheet. Data categories will follow the research questions presented in the Introduction. Data will be extracted on the geographical scope, extent, and resolution of studies. The temporal scale of the research studies will be coded either as historical, present, or future. Conservation features, their numbers, taxonomic groups, and biome addressed will also be extracted. The time span of studies will not be reported, as based on the piloted data extraction (S1 Table), years are not necessarily reported.

The threat categories, the number of threats addressed in the study, and threat typologies will be reported. Data and methodological approaches for threats will also be coded. Most importantly, the approach to using threat information in conservation plans, will be extracted.

For studies that do not cite scientific literature on the theory of threats, or evidence of threats impacting biodiversity features, expert knowledge is assumed. Authors will not be contacted regarding missing data. For studies that develop threat data based on external models or analysis outside the research article itself, external references are separately extracted and coded.

Extracted threats will be classified according to the IUCN Threat classification scheme (version 3.2). All human-induced threats will be covered, and where a threat cannot be assigned to a certain category, the class “Other” will be used.

The functioning of data sheets was piloted with the selected 12 benchmark articles, summarized in S1 Table. A random 10% of the articles will be chosen for data extraction by another reviewer. Should disagreements arise, the data extraction methodology will be clarified.

### Data synthesis and presentation

This study will synthetize information from eligible studies in a systematic review. A database of literature reviewed together with coded metadata will be included as supplementary data. Summary statistics on extracted data will be included, based on biome, resolution, biodiversity features, scale of studies, and methodological approaches utilized. Gaps in knowledge about threats in conservation plans are summarized and visualized, and as far as the data extracted allows, guidelines are provided on future reporting.

## Supporting information

S1 File PRISMA and ROSES sheets. (DOCX)

S2 File Scoping search for keywords. (DOCX)

S3 File Search plan for keywords and syntaxes. (DOCX)

S4 File List of benchmark articles. (DOCX)

S1 Table Piloted data extraction strategy. (XLSX)
