# Supplementary material for: How threats inform conservation planning—A systematic review protocol
Source: PLoS One. 2022 May 31;17(5):e0269107. doi: 10.1371/journal.pone.0269107 (PMC9154108; doi:10.1371/journal.pone.0269107)
Supplement: S2 File — (DOCX) [file pone.0269107.s002.docx]

**Supporting file 2. Scoping phase searches to define keywords and search strategy**

| **Database and date** | **Search procedure and syntax**  **TS=Topic search** | **Result** | **Limitations** |
| --- | --- | --- | --- |
| 1^st^ search in Web of Science  28.10.21 | TS=("systematic conservation plan*" OR "conservation plan*" OR "spatial conservation prioritization" OR "planning protected area*" OR "planning conservation area*" OR "spatial prioritization") AND TS= (threat* OR pressure* OR "human activ*" OR stressor* OR "human impact*") | 2709 | No |
| 2^nd^ search in Web of Science  29.10.21-1.11.21 | TS=(“protected area*” OR conservation area*) AND TS=( threat* OR pressure* OR stressor*) AND TS=(plan* OR design* OR propos*) | 10408 | No |
| 3^rd^ search in Web of Science  29.10.21-1.11.21 | TS=("protected area*") AND TS=(conservation area*) AND TS=("human activ*" OR "human impact") AND TS=(plan* OR design* OR propos*) | 532 | No |
| 4^th^ search in Web of Science  29.10.21-1.11.21 | TS=((“conservation area*”) NEAR/10 ( threat* OR pressure* OR stressor* OR “human activ*” OR “human impact*”)) | 150 | No |
| 5^th^ search in Web of Science  29.10.21-1.11.21 | TS=((“protected area*”) NEAR/10 (plan*)) AND TS=(threat* OR pressure* OR stressor* "human activ*” OR "human impact") | 185 | No |
| 6^th^ search in Web of Science  29.10.21-1.11.21 | TS=(“protected area*”) AND TS=( conservation area*) AND TS=( threat* OR pressure* OR stressor*) AND TS=(plan* OR design* OR propos*) | 200 | No |
| 7^th^ search in Web of Science  29.10.21-1.11.21 | TS=((“conservation area*” OR "protected area*") NEAR/5 ( threat* OR pressure* OR stressor* OR “human activ*” OR “human impact*”)) | 874 | No |
| 8^th^ search in Web of Science  29.10.21-1.11.21 | TS=(("conservation plan*" OR "protected area*") NEAR/5 (threat* OR pressure* OR stressor* OR “human activ*" OR “human impact*”)) | 1005 | No |
| 9^th^ search in Web of Science  29.10.21-1.11.21 | TS=("conservation plan*" OR “conservation priorit*” OR "conservation area*" OR "spatial priorit*") AND TS= (threat* OR pressure* OR stressor* OR "human activ*” OR "human impact*") | 4369 | No |
| 10^th^ search in Web of Science  2.11.21 | TS=("spatial prioritization*" OR “conservation priorit*" OR “systematic conservation”) AND TS=(threat* OR pressure* OR stressor* OR anthropogenic OR risk* OR impact*) | 2559 | No |
| 11^th^ search in Web of Science  2.11.21 | TS=(“protected area*” OR "conservation area*") AND TS=(threat* OR pressure* OR stressor* OR anthropogenic OR risk* OR impact*) AND TS=(plan OR plans OR planning) | 3332 | No |
| 11^th^ search in Web of Science  2.11.21 | TS=("conservation planning" OR "conservation plans" OR "conservation plan") AND TS=(threat* OR pressure* OR stressor* OR anthropogenic OR risk* OR impact*) | 4134 | No |
| 12^th^ search in Web of Science  21.12.21 | TS=(”reserve selection”) | 781 | No |
| 13^th^ search in Web of Science  21.12.21 | TS=("reserve selection") AND TS=(threat* OR pressure* OR stressor* OR anthropogenic OR risk* OR impact*) | 274 | No |
| 14^th^ search in Web of Science  21.12.21 | TS=(”site selection”) | 14776 | No |
| 15^th^ search in Web of Science  21.12.21 | TS=("site selection”) AND TS=(threat* OR pressure* OR stressor* OR anthropogenic OR risk* OR impact*) | 3875 | No |
| 16^th^ search in Web of Science  21.12.21 | TS=("site selection") AND TS=(threat* OR pressure* OR stressor* OR anthropogenic OR risk* OR impact*) AND TS=(planning OR plan OR plans OR planner* OR conservation OR conserving OR conserve OR protection OR protecting OR preservation OR restoration) | 1461 | No |
| 17^th^ search in Web of Science  11.1.2022 | TS=("site selection") AND TS=(threat* OR pressure* OR stressor* OR anthropogenic OR risk* OR impact*) AND TS=(plan OR plans OR planning OR planned OR design OR designs OR designing OR designed OR conservation OR conserving OR conserve OR protection OR protecting OR preservation) | 1710 | No |
| 18^th^ search in Web of Science  11.1.2022 | TS=("site selection") AND TS=(threat* OR pressure* OR stressor* OR anthropogenic OR risk* OR impact*) | 3889 | No |
| 19^th^ search in Web of Science  11.1.2022 | TS=("site selection") AND TS=(threat* OR pressure* OR stressor* OR anthropogenic OR risk* OR impact*) AND TS=(conservation OR conserving OR conserve OR protection OR protecting OR preservation) | 1100 | No |
| 20^th^ search in Web of Science  24.1.2022 | TS=("conservation plan*" OR "spatial prioritization*" OR “conservation priorit*" OR “systematic conservation” OR “reserve selection”) AND TS=(threat* OR pressure* OR stressor* OR anthropogenic OR risk* OR impact*) | 6322 | No |
