# Supplementary material for: How threats inform conservation planning—A systematic review protocol
Source: PLoS One. 2022 May 31;17(5):e0269107. doi: 10.1371/journal.pone.0269107 (PMC9154108; doi:10.1371/journal.pone.0269107)
Supplement: S3 File — (DOCX) [file pone.0269107.s003.docx]

**Supporting file 3. Keywords, search steps and a plan for conducting the searches**

**Keywords**

| Conservation planning terms | conservation plan, conservation planning, conservation prioritization, reserve selection, site selection, spatial prioritization, systematic conservation |
| --- | --- |
| Conservation area terms | conservation area, protected area |
| Threat and related terms | threat, pressure, stressor, anthropogenic, risk, impact |
| Planning terms | planning, plan, design, designing |
| Conservation terms | conservation, conserving, protection, protecting, preservation |

**Search steps and syntaxes**

|  |  |
| --- | --- |
| Step 1  Topic: Conservation planning | ("conservation plan*" OR "spatial prioritization*" OR “conservation priorit*" OR “systematic conservation” OR "reserve selection") AND (threat* OR pressure* OR stressor* OR anthropogenic OR risk* OR impact*) |
| Step 2  Topic: Conservation area | ("conservation area*" OR “protected area*”) AND (threat* OR pressure* OR stressor* OR anthropogenic OR risk* OR impact*) AND (plan OR plans OR planning OR planned OR design OR designs OR designing OR designed) |
| Step 3  Topic: Site selection | ("site selection") AND (threat* OR pressure* OR stressor* OR anthropogenic OR risk* OR impact*) AND (plan OR plans OR planning OR planned OR design OR designs OR designing OR designed OR conservation OR conserving OR conserve OR protection OR protecting OR preservation) |

**A plan for conducting the searches**

| **Database and date** | **Search procedure and syntax** | **Result** | **Limitations** |
| --- | --- | --- | --- |
| **Search step 1: Conservation planning** | | | |
| Web of Science | Title, Abstract, Author keyword searching  (TI=("conservation plan*" OR "spatial prioritization*" OR “conservation priorit*" OR “systematic conservation” OR "reserve selection") OR AB=("conservation plan*" OR "spatial prioritization*" OR “conservation priorit*" OR “systematic conservation” OR "reserve selection") OR AK=("conservation plan*" OR "spatial prioritization*" OR “conservation priorit*" OR “systematic conservation” OR "reserve selection")) AND (TI=(threat* OR pressure* OR stressor* OR anthropogenic OR risk* OR impact*) OR AB=(threat* OR pressure* OR stressor* OR anthropogenic OR risk* OR impact*) OR AK=(threat* OR pressure* OR stressor* OR anthropogenic OR risk* OR impact*) OR AK=(threat* OR pressure* OR stressor* OR anthropogenic OR risk* OR impact*)) |  | No |
| Scopus | Title, Abstract, Author keyword searching  TITLE-ABS-KEY("conservation plan*" OR "spatial prioritization*" OR “conservation priorit*" OR “systematic conservation” OR "reserve selection") AND TITLE-ABS-KEY(threat* OR pressure* OR stressor* OR anthropogenic OR risk* OR impact*) |  | No |
| **Search step 2: Conservation area** | | | |
| Web of Science | (TI=(“conservation area*” OR " protected area*") OR AB=(“conservation area*” OR " protected area*") OR AK=(“conservation area*” OR " protected area*")) AND (TI=(threat* OR pressure* OR stressor* OR anthropogenic OR risk* OR impact*) OR AB=(threat* OR pressure* OR stressor* OR anthropogenic OR risk* OR impact*) OR AK=(threat* OR pressure* OR stressor* OR anthropogenic OR risk* OR impact*)) AND (TI=(plan OR plans OR planning OR planned OR design OR designs OR designing OR designed) OR AB=(plan OR plans OR planning OR planned OR design OR designs OR designing OR designed) OR AK=( plan OR plans OR planning OR planned OR design OR designs OR designing OR designed)) |  | No |
| Scopus | Title, Abstract, Author keyword searching  TITLE-ABS-KEY(“conservation area*” OR " protected area*") AND (threat* OR pressure* OR stressor* OR anthropogenic OR risk* OR impact*) OR AB=(threat* OR pressure* OR stressor* OR anthropogenic OR risk* OR impact*) OR AK=(threat* OR pressure* OR stressor* OR anthropogenic OR risk* OR impact*) AND TITLE-ABS-KEY(plan OR plans OR planning OR planned OR design OR designs OR designing OR designed) |  | No |
| **Search step 3: Site selection** | | | |
| Web of Science | Title, Abstract, Author keyword searching  (TI=("site selection") OR AB=("site selection") OR AK=("site selection")) AND (TI=(threat* OR pressure* OR stressor* OR anthropogenic OR risk* OR impact*) OR AB=(threat* OR pressure* OR stressor* OR anthropogenic OR risk* OR impact*) OR AK=(threat* OR pressure* OR stressor* OR anthropogenic OR risk* OR impact*)) AND (TI=(plan OR plans OR planning OR planned OR design OR designs OR designing OR designed OR conservation OR conserving OR conserve OR protection OR protecting OR preservation) OR AB=(plan OR plans OR planning OR planned OR design OR designs OR designing OR designed OR conservation OR conserving OR conserve OR protection OR protecting OR preservation) OR AK=(plan OR plans OR planning OR planned OR design OR designs OR designing OR designed OR conservation OR conserving OR conserve OR protection OR protecting OR preservation)) |  | No |
| Scopus | Title, Abstract, Author keyword searching  TITLE-ABS-KEY("site selection") AND TITLE-ABS-KEY(threat* OR pressure* OR stressor* OR anthropogenic OR risk* OR impact*) AND TITLE-ABS-KEY(plan OR plans OR planning OR planned OR design OR designs OR designing OR designed OR conservation OR conserving OR conserve OR protection OR protecting OR preservation) |  | No |
