# Supplementary material for: How threats inform conservation planning—A systematic review protocol
Source: PLoS One. 2022 May 31;17(5):e0269107. doi: 10.1371/journal.pone.0269107 (PMC9154108; doi:10.1371/journal.pone.0269107)
Supplement: S4 File — (DOCX) [file pone.0269107.s004.docx]

**Supporting file 4. List of benchmark articles**

Assessing search strategy comprehensiveness using benchmark articles

| **Research article** | | **DOI** | **Web of Science / Search steps for A plan to conduct searches** | | |
| --- | --- | --- | --- | --- | --- |
|  |  |  | Search step 1:  Conservation planning | Search step 2:  Conservation area | Search step 3:  Site selection |
| 1 | Foresta M, Carranza ML, Garfì V, Di Febbraro M, Marchetti M, Loy A. A systematic conservation planning approach to fire risk management in Natura 2000 sites. Journal of environmental management. 2016;181:574-81. | <https://doi.org/10.1016/j.jenvman.2016.07.006> | Yes | Yes | No |
| 2 | Fraschetti, Simonetta, Paolo D’Ambrosio, Fiorenza Micheli, Fausto Pizzolante, Simona Bussotti, and Antonio Terlizzi. Design of marine protected areas in a human-dominated seascape. Marine Ecology Progress Series. 2009; 375: 13-24. | <https://doi.org/10.3354/meps07781> | Yes | Yes | Yes |
| 3 | Green A, Smith SE, Lipsett-Moore G, Groves C, Peterson N, Sheppard S, et al. Designing a resilient network of marine protected areas for Kimbe Bay, Papua New Guinea. Oryx. 2009;43(4):488-98. | <https://doi.org/10.1017/S0030605309990342> | Yes | Yes | No |
| 4 | Lira-Noriega A, Aguilar V, Alarcón J, Kolb M, Urquiza-Haas T, González-Ramírez L, et al. Conservation planning for freshwater ecosystems in Mexico. Biological Conservation. 2015;191:357-66. | <https://doi.org/10.1016/j.biocon.2015.07.004> | Yes | Yes | No |
| 5 | Mizsei E, Szabolcs M, Szabó L, Boros Z, Mersini K, Roussos SA, et al. Determining priority areas for an Endangered cold-adapted snake on warming mountaintops. Oryx. 2021;55(3):334-43. | https://doi.org/10.1017/S0030605319000322 | Yes | Yes | No |
| 6 | Lawrence DJ, Larson ER, Liermann CAR, Mims MC, Pool TK, Olden JD. National parks as protected areas for US freshwater fish diversity. Conservation Letters. 2011;4(5):364-71. | <https://doi.org/10.1111/j.1755-263X.2011.00185.x> | Yes | Yes | No |
| 7 | Almpanidou V, Doxa A, Mazaris AD. Combining a cumulative risk index and species distribution data to identify priority areas for marine biodiversity conservation in the Black Sea. Ocean & Coastal Management. 2021;213:105877. | <https://doi.org/10.1016/j.ocecoaman.2021.105877> | Yes | Yes | No |
| 8 | Carrasco J, Price V, Tulloch V, Mills M. Selecting priority areas for the conservation of endemic trees species and their ecosystems in Madagascar considering both conservation value and vulnerability to human pressure. Biodiversity and Conservation. 2020;29(6):1841-54. | <https://doi.org/10.1007/s10531-020-01947-1> | Yes | No | No |
| 9 | Junker J, Boesch C, Freeman T, Mundry R, Stephens C, Kühl HS. Integrating wildlife conservation with conflicting economic land-use goals in a West African biodiversity hotspot. Basic and Applied Ecology. 2015;16(8):690-702. | <https://doi.org/10.1016/j.baae.2015.07.002> | Yes | Yes | No |
| 10 | Zhang M-G, Slik JF, Ma K-P. Priority areas for the conservation of perennial plants in China. Biological Conservation. 2017;210:56-63. | <https://doi.org/10.1016/j.biocon.2016.06.007> | Yes | No | No |
| 11 | Tognelli, Marcelo F., Agustín M. Abba, J. Benjamín Bender, and Viviana P. Seitz. Assessing conservation priorities of xenarthrans in Argentina. Biodiversity and Conservation. 2011; 20(1): 141-151. | <https://doi.org/10.1007/s10531-010-9951-5> | Yes | Yes | No |
| 12 | Prieto-Torres DA, Nori J, Rojas-Soto OR. Identifying priority conservation areas for birds associated to endangered Neotropical dry forests. Biological Conservation. 2018;228:205-14. | <https://doi.org/10.1016/j.biocon.2018.10.025> | Yes | Yes | No |
